# Global comparison of cancer outcomes: standardization and correlation with healthcare expenditures

**DOI:** 10.1186/s12889-019-7384-y

**Published:** 2019-08-07

**Authors:** Horace C. W. Choi, Ka-on Lam, Herbert H. M. Pang, Steven K. C. Tsang, Roger K. C. Ngan, Anne W. M. Lee

**Affiliations:** 1 Department of Clinical Oncology, Li Ka Shing Faculty of Medicine, The University of Hong Kong, Hong Kong, China; 2 Clinical Oncology Center, The University of Hong Kong-Shenzhen Hospital, Shenzhen, China; 3 School of Public Health, Li Ka Shing Faculty of Medicine, The University of Hong Kong, Hong Kong, China

**Keywords:** Global health, Cancer site-standardized relative survival, Cancer outcomes, Health economics

## Abstract

**Background:**

Cancer outcomes vary widely among different countries. However, comparisons of cost-effectiveness and cost-efficiency of different systems are complex because the incidences of different cancers vary across countries and their chances of cure also differ substantially. We aim to propose a new standardized method for global comparison and to explore its relationship with economic indicators.

**Methods:**

Cancer statistics from all 184 countries and 27 cancers listed in GLOBOCAN 2012 were analyzed. The complement of age-standardized mortality/incidence ratio [1 – (ASM/ASI)] was taken as the proxy relative survival (RS). Accounting for various country-specific cancer patterns, the cancer site-standardized proxy RS (proxy SS-RS) of individual countries were calculated by weighting the proportion of specific cancer sites as compared with the global pattern of incidence. Economic indicators of different countries listed by the World Bank were correlated with corresponding proxy SS-RS.

**Results:**

Substantial variation in site-specific survival and new case distribution supported the use of proxy SS-RS, which ranged from 0.124 to 0.622 (median 0.359). The median total health expenditure per capita (HEpc) increased from US$44 for countries with proxy SS-RS < 0.25, to US$4643 for countries with proxy SS-RS ≥0.55. Results from logarithmic regression model showed exponential increase in total HEpc for better outcome. The expenditure varied widely among different strata, with the widest difference observed among countries with SS-RS ≥0.55 (total HEpc US$1412–$9361).

**Conclusions:**

Similar to age-standardization, cancer site-standardization adjusted for variation in pattern of cancer incidence provides the best available and feasible strategies for comparing cancer survivals across countries globally. Furthermore, cancer outcome correlated significantly with economic indicators and the amount of HEpc escalated exponentially. Our findings call for more in-depth studies applying cancer-site standardization to provide essential data for sharing of experience and urgent actions by policy makers to develop comprehensive and financially sustainable cancer plan for greater equity.

**Electronic supplementary material:**

The online version of this article (10.1186/s12889-019-7384-y) contains supplementary material, which is available to authorized users.

## Background

Cancer is a major health burden across all countries and the problem is rapidly escalating. According to the GLOBOCAN 2012 by the International Agency for Research on Cancer (IARC), there were 14.1 million new cancer cases (all cancers excluding non-melanoma skin cancers) and a total of 8.2 million cancer deaths globally in the year 2012 [1]. In the World Cancer Report 2014, it was projected that the incidence and mortality would continue to rise to 22 million and 13 million, respectively, in the coming two decades with more than 57% of the overall cancer incidence and 65% of mortality occurring in less developed regions [2].

Cancer outcomes vary widely among different countries due to multiple factors including variation in pattern of cancers, national cancer screening policy, presenting stage, access to good quality treatment (e.g., radiotherapy and systemic therapy), and cultural barriers. The World Health Assembly Resolution on Cancer stated that closing the cancer divide on all fronts was a pressing global health issue and called upon all governments to implement plans for reducing cancer mortality [3]. Assessment of outcomes data is fundamental for gauging the results achieved by individual country and goal-setting the target achievable with contemporary provision. However, this is not straightforward because many cancer registries, especially those in less developed regions, do not have prospectively collected individual patient record for calculation of relative survival (RS) [1]. One acceptable surrogate for RS is to use the complement of the ratio of age-standardized mortality/incidence rate [1 – (ASM/ASI)] and this is supported by the study by Vostakolaei et al. on 32 cancer sites in seven Western countries [4].

Marked variation exists in the incidence patterns of different cancers and thus the chance of cure (even with best contemporary treatment) differs substantially. For instance, a country with high incidence of good-prognosis cancer may have a better overall RS than another country with high incidence of poor-prognosis cancer, confounding the interpretations of their actual standard of cancer care. Adjustment for these variations is particularly important for a fair comparison of the cost-efficiency (i.e. the maximum possible health outcomes / benefits given the same amount of resources) of different health systems among different countries. Therefore, in the current study we introduced the concept of the cancer site-standardized proxy RS (proxy SS-RS) to enable an unbiased comparison across the globe.

In addition, survival for cancer patients depends on access to appropriate treatment, which in term is closely linked to the economic capability and healthcare policy of individual countries. Since there is very little global data on detailed breakdown of the national expenditure specific for cancer treatment, we analysed the correlation between the overall expenditure of different countries on healthcare and their respective cancer outcome in order to provide useful reflection on the essential requirement and the cost-efficiency of different healthcare systems worldwide.

In the current study, we standardized the proxy RS by cancer sites and compare the values across different countries. The corresponding correlation of proxy SS-RS with national economic indicators were also analysed. The aims were to provide a tool for unbiased comparison of outcomes in different countries, and to provide standardized outcome indicators for estimating the health expenditures needed to achieve the respective target outcomes.

## Methods

### Sources of data

The cancer outcome data were based on the GLOBOCAN 2012 [1]: age-standardized rates of incidence (ASI) and mortality (ASM) from all 184 countries/regions were retrieved. Both the overall rates for all cancers excluding non-melanoma skin cancer and the specific rates for the 27 listed cancer sites were studied. In GLOBOCAN 2012, rates for non-melanoma skin cancer were not available because many of such cancers were never diagnosed and were under-recorded in cancer registry [1].

The economic data were based on the statistics released by The World Bank Group [5]: indicators including gross national income (GNI), gross domestic product (GDP), total health expenditure (total HE), and health expenditure in public sector (public HE) were retrieved, in which monetary values per capita (pc) were used and were denominated in US dollars.

In addition to data of individual countries, the following categorizations were studied: continents, development regions (more / less developed, as defined in the GLOBOCAN 2012), and the Human Development Index (HDI) introduced by Human Development Reports [6] (with countries categorized into “low”, “medium”, “high” and “very high” development tiers basing on life expectancy at birth, mean and expected years of schooling, and GNI per capita). To be consistent with the GLOBOCAN 2012 outcome data, all the economic indicators and the HDI values and corresponding tiers were based on the data recorded for the year 2012.

### Statistical analysis

The proxy RS for each country was calculated using the formula [1 – (ASM/ASI)]. Cancer site-specific proxy RS and patterns of incidence were also presented. Furthermore, the proxy SS-RS were calculated to adjust for the variation in incidence of different cancers. The concept and the methodology used were analogous to that used for age-standardization for adjustment to variation in age distribution [7]. The cancer incidence in the World population as shown by GLOBOCAN 2012 were used as the “standard”, the proxy SS-RS for each country was calculated by the formula: ∑_*i*_(*r*_*i*_ × *p*_*i*_); that is, the summation of proxy RS of each cancer site *i* achieved by individual country (*r*_*i*_) × corresponding proportion of that specific cancer site in the world population (*p*_*i*_). Additional file 1: Table S1 demonstrates an example on calculation of proxy SS-RS. Comparisons of proxy SS-RS between subgroups in categorizations (continents, more / less developed and HDI tiers) were performed by Mood’s median test because the proxy RS might not be normally distributed.

The range of economic indicators across countries at various range of proxy SS-RS were presented. Regarding the correlation of SS-RS with economic indicators, fractional polynomials was used to assess potential transformation [8]. Taking into account of models’ residual deviance and comparing against linear, square or cubic transformations, fractional polynomials suggested that logarithmic transformation over economic indicators would generate the best fits to proxy SS-RS. Hence, logarithmic regression model was applied to estimate the monetary values needed to achieve a particular proxy SS-RS target, with the natural logarithm (*ln*, base *e*) of the economic indicators as the independent factor. The goodness-of-fit using the adjusted R-squared (R^2^) statistic was used to identify the economic indicators which showed the strongest correlation (i.e., the indicators with the highest adjusted R^2^).

All statistical analyses were conducted using R version 3.2.5 (R Core Team, Vienna, Austria) with fractional polynomials model fitting using mfp package, and map plotting using maps and mapdata packages. We considered *P* < 0.05 as statistically significant.

## Results

### Unadjusted proxy RS

**Fig. 1:**
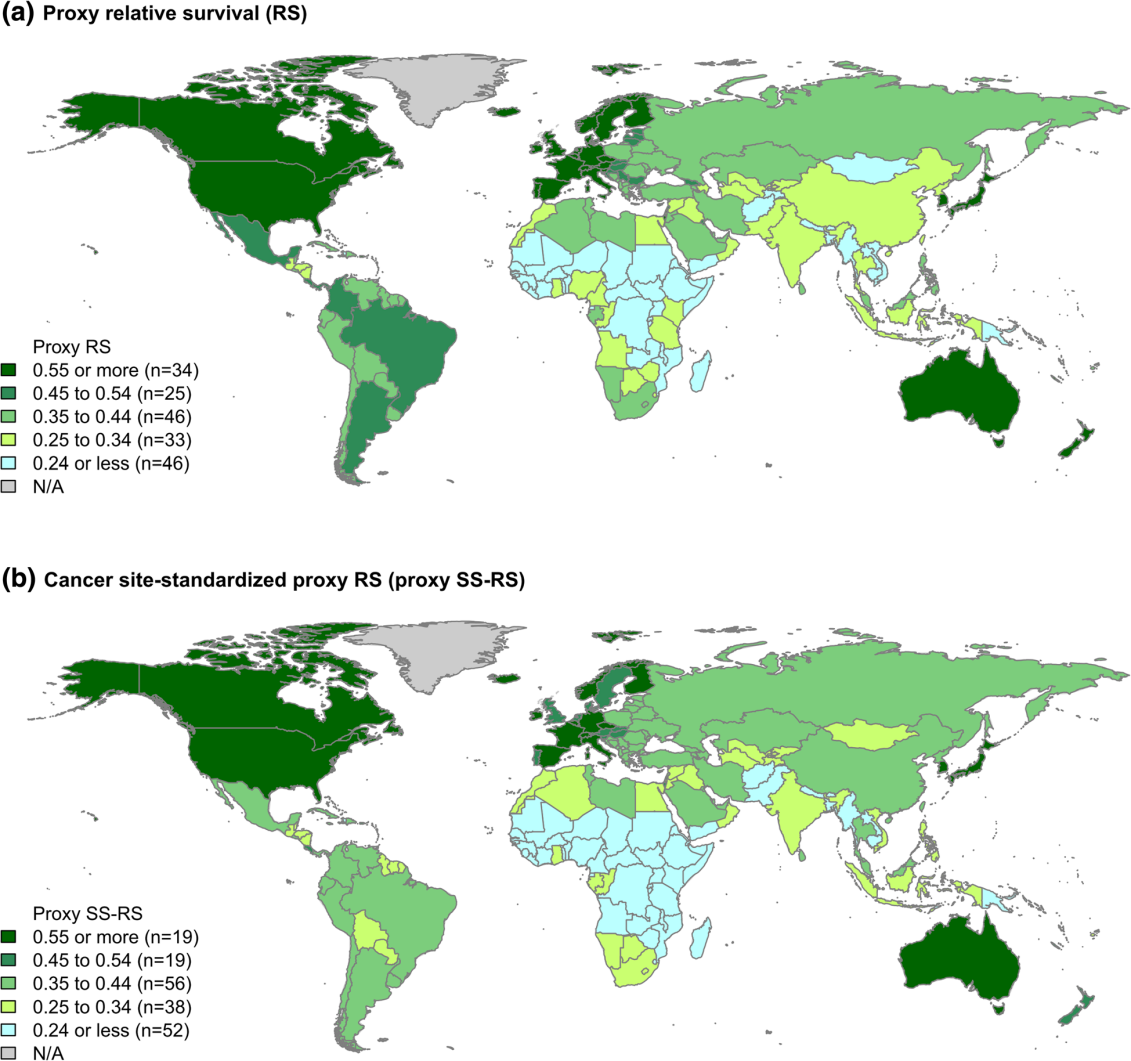
**a** Proxy relative survival (RS) / **b** cancer site-standardized proxy RS by countries (GLOBOCAN 2012)

The global pattern of proxy RS (Fig. 1a) was different from that of proxy SS-RS (Fig. 1b) among the 184 countries studied; the proxy RS of different countries varied widely from 0.111 to 0.702 (median = 0.386). Altogether, 34 (18%) countries achieved proxy RS ≥0.55 while 46 (25%) countries had proxy RS < 0.25. Table 1 shows the outcomes and economic indicators in countries that achieved proxy RS ≥0.55. The top 3 countries with the highest unadjusted proxy RS were Australia (0.702), Iceland (0.692) and Norway (0.688).

**Table 1:** Proxy relative survival (RS), cancer site-standardized proxy RS and health expenditures (GLOBOCAN 2012) among countries with proxy RS ≥0.55

Rank	Country ^a^	No. of new cases ^b^	Unadjusted proxy RS	Site-standardized proxy RS (rank)	Total health expenditure per capita (US$) ^c^	Public health expenditure per capita (US$) ^c^
1	Australia	122,031	0.702	0.609 (2)	6543.5	4395.5
2	Iceland	1449	0.692	0.586 (5)	3856.8	3106.5
3	Norway	28,214	0.688	0.594 (3)	9360.8	7947.0
4	Switzerland	42,046	0.678	0.586 (6)	9195.7	5950.1
5	Republic of Korea	219,520	0.674	0.622 (1)	1714.9	941.9
6	USA	1,603,586	0.667	0.587 (4)	8789.8	4154.0
7	Israel	29,176	0.667	0.536 (24)	2514.6	1581.8
8	Finland	28,428	0.665	0.559 (16)	4254.8	3221.8
9	Puerto Rico	11,822	0.663	0.550 (19)	n.a.	n.a.
10	Sweden	50,481	0.659	0.521 (28)	6521.6	5501.1
11	Luxembourg	2476	0.654	0.579 (9)	7550.7	6301.6
12	Canada	182,182	0.651	0.579 (8)	5719.0	4059.4
13	New Zealand	21,337	0.648	0.539 (23)	4470.9	3702.1
14	Ireland	20,808	0.648	0.561 (15)	4079.5	2756.6
15	Germany	493,780	0.645	0.583 (7)	4753.9	3616.1
16	France	349,426	0.644	0.574 (11)	4698.9	3625.6
17	Belgium	65,345	0.638	0.564 (14)	4587.9	3563.5
18	Italy	354,456	0.635	0.552 (17)	3242.2	2442.7
19	Malta	1902	0.632	0.567 (13)	2216.6	1475.7
20	Denmark	36,119	0.631	0.544 (21)	6203.8	5320.3
21	Cyprus	3438	0.617	0.518 (29)	1964.8	901.1
22	The Netherlands	93,448	0.616	0.529 (26)	5456.5	4721.2
23	France, Martinique	1808	0.612	0.444 (41)	n.a.	n.a.
24	Spain	215,534	0.606	0.571 (12)	2651.4	1901.8
25	Portugal	49,174	0.598	0.542 (22)	1999.8	1280.5
26	United Kingdom	327,812	0.597	0.534 (25)	3648.7	3025.2
27	Austria	41,117	0.593	0.549 (20)	5239.5	3948.9
28	Czech Republic	57,627	0.586	0.551 (18)	1411.5	1185.9
29	Barbados	1144	0.585	0.429 (46)	1137.9	741.6
30	Slovenia	11,457	0.577	0.512 (32)	2068.5	1502.5
31	New Caledonia	886	0.573	0.470 (35)	n.a.	n.a.
32	Japan	703,863	0.568	0.577 (10)	4748.9	3927.1
33	France, La Reunion	1868	0.564	0.517 (30)	n.a.	n.a.
34	Singapore	15,693	0.562	0.529 (27)	2310.4	821.3

Data sources: GLOBOCAN 2012 for the calculation of number of new cases, unadjusted proxy RS and cancer site-standardized proxy RS;^1^ The World Bank for health expenditures^5^

^a^ Countries with unadjusted proxy RS ≥0.55 were listed

^b^ Refer to the number of new cases for “all cancers excluding non-melanoma of skin” ^1^

^c^ n.a.: Total and public health expenditures were not available for these countries for the year 2012^5^

Table 2 shows the site-specific proxy RS of the 27 cancer sites listed and the marked variation in distribution among the 184 countries. Cancers of thyroid, testis, corpus uteri, and melanoma of skin achieved the best outcome with site-specific proxy RS ranging from 0.767 to 0.875. The survival outcomes were the worst for cancers of pancreas, liver, lung, and oesophagus with median proxy RS ranging from 0.048 to 0.153; these four cancer sites attributed to 24.1% of the overall new caseload in 2012 globally. Furthermore, the cancers having the widest variation in distribution could have markedly different outcomes. The proportion of new cases attributed to prostate cancer ranged from 0.6% in Democratic Republic of Korea to 42.6% in Martinique (France) and that proportion of liver cancer varied similarly from 0.5% in the Netherlands to 42.7% in Gambia. However, the median proxy RS among individual countries showed differently at 0.745 for prostate cancer and at 0.059 for liver cancer.

**Table 2:** Proxy relative survival (RS) and distribution of the 27 cancer sites (GLOBOCAN 2012)

Cancer sites	World population		Proportion (%) of new cases among different countries	
	Proxy RS	Proportion (%) of new cases ^a^	Range	Difference ^b^
Thyroid	0.875	2.1	(0.0, 15.0)	15.0
Testis	0.800	0.4	(0.0, 1.6)	1.6
Corpus uteri	0.780	2.3	(0.0, 6.6)	6.6
Melanoma of skin	0.767	1.7	(0.0, 11.6)	11.6
Prostate	0.745	7.8	(0.6, 42.6)	42.0
Breast	0.701	11.9	(2.8, 26.7)	23.9
Hodgkin lymphoma	0.667	0.5	(0.0, 3.7)	3.7
Bladder	0.642	3.1	(0.2, 8.6)	8.4
Kidney	0.591	2.4	(0.0, 5.7)	5.7
Lip, oral cavity	0.525	2.1	(0.0, 13.6)	13.6
Colorectum	0.517	9.7	(0.7, 17.0)	16.3
Cervix uteri	0.514	3.8	(0.5, 32.7)	32.2
Non-Hodgkin lymphoma	0.500	2.7	(0.4, 12.0)	11.6
Larynx	0.476	1.1	(0.0, 4.1)	4.1
Nasopharynx	0.417	0.6	(0.0, 5.4)	5.4
Ovary	0.393	1.7	(0.4, 5.7)	5.3
Kaposi sarcoma	0.333	0.3	(0.0, 26.2)	26.2
Multiple myeloma	0.333	0.8	(0.0, 2.8)	2.8
Other pharynx	0.316	1.0	(0.0, 8.1)	8.1
Leukaemia	0.277	2.5	(0.0, 10.0)	10.0
Brain, nervous system	0.265	1.8	(0.0, 5.7)	5.7
Stomach	0.264	6.8	(0.5, 20.0)	19.5
Gallbladder	0.227	1.3	(0.0, 5.6)	5.6
Oesophagus	0.153	3.2	(0.0, 12.8)	12.8
Lung	0.147	13.0	(0.4, 25.0)	24.6
Liver	0.059	5.6	(0.5, 42.7)	42.2
Pancreas	0.048	2.4	(0.0, 4.7)	4.7

^a^ Proportion of new cases among all cancers except non-melanoma skin cancer

^b^ Difference in proportion of new cases between the countries with the highest and lowest proportions

**Fig. 2:**
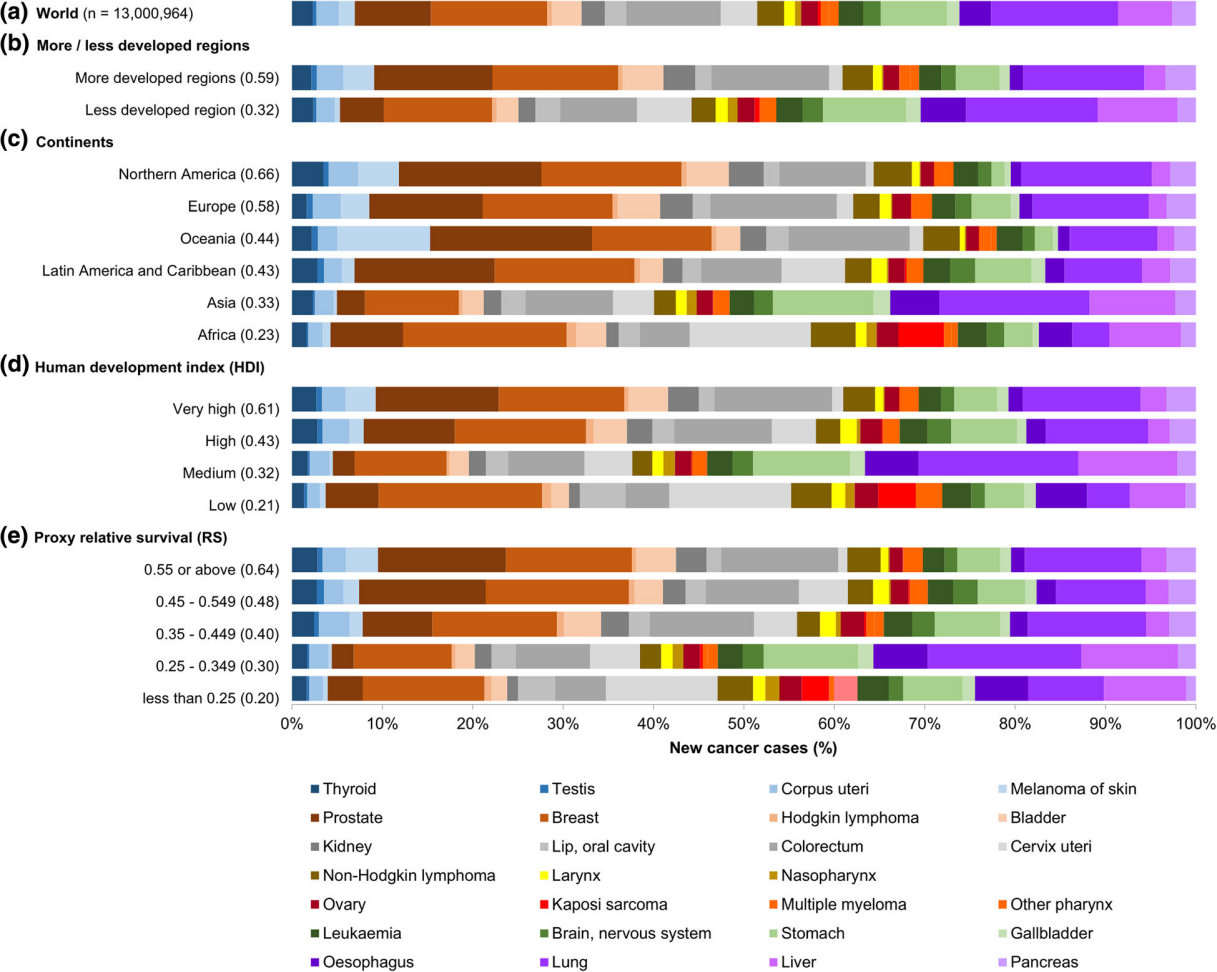
Distribution of new cancer cases by country profiles (GLOBOCAN 2012). The cancers were ordered in descending proxy relative survival (RS). Note: Parentheses after categories present the median proxy RS among the countries attributed in that corresponding categories

Figure 2 shows the distribution of new case incidence of different cancer sites by different categorizations. There were higher proportion of prostate and colorectal cancers in more developed regions, high and very high HDI tiers, and countries with proxy RS ≥0.35. In contrast, cancers of oesophagus, liver and cervix were more common in less developed regions, low and medium HDI tiers, and countries with proxy RS <0.35. By continents, prostate cancer was comparatively more common in Oceania, Northern America, Latin America and Caribbean, and Europe; while liver and cervix cancers were more common in Africa and Asia. Furthermore, melanoma of skin was uniquely common in Oceania. The substantial variations in both survival outcomes and incidence distributions suggest the need of appropriate standardization for a balanced outcome measure for comparison across the globe.

### Cancer site-standardized proxy RS

Figure 1b shows the global pattern of proxy SS-RS adjusted for the marked variation in distribution of different cancer sites. The proxy SS-RS of 184 countries ranged from 0.124 to 0.622 (median = 0.359). Approximately 10% of the countries achieved proxy SS-RS ≥0.55 (*n* = 19) and in the range 0.45–<0.55 (*n* = 19). The top 3 countries with the highest proxy SS-RS were Republic of Korea (0.622), Australia (0.609) and Norway (0.594) (Table 1). Compared with the unadjusted proxy RS, the relative change of cancer proxy SS-RS ranged from − 0.168 (decrease) to + 0.098 (increase) unit.

**Fig. 3:**
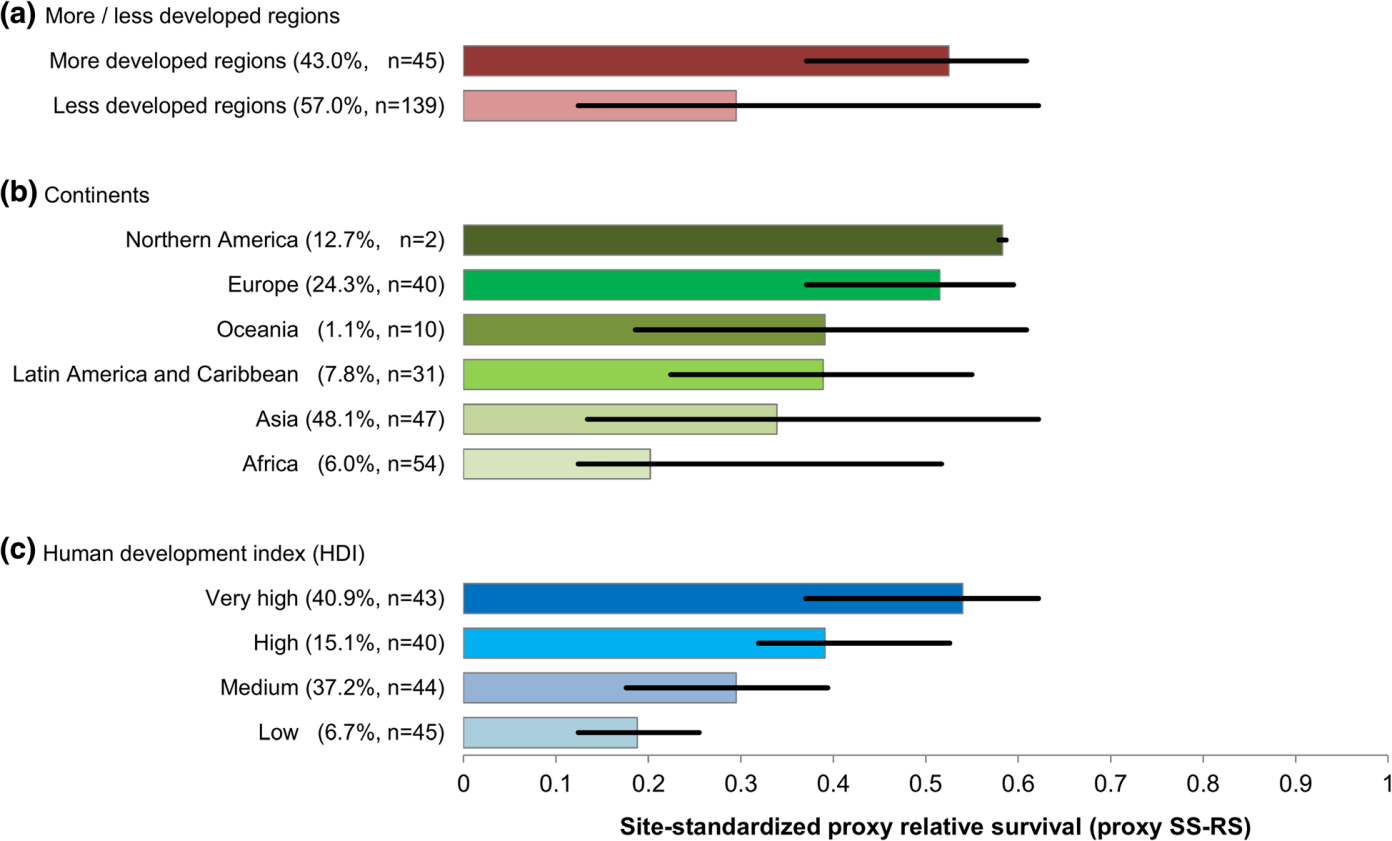
Site-standardized proxy relative survival (proxy SS-RS) by country profiles. Values in parentheses indicate (i) the proportion of the incidence among all new cancer cases (except non-melanoma skin cancer for each cancer site) and (ii) the number of countries in each subcategory. The bars represented the median proxy SS-RS and the strokes represented the corresponding minimum and maximum proxy SS-RS among the countries within each subcategory

Figure 3 shows the proxy SS-RS by different categorizations. The proxy SS-RS was significantly higher in more developed regions compared with less developed regions (median, 0.525 vs 0.295, *P* < 0.001). By continents, the median proxy SS-RS ranged from 0.583 in Northern America, 0.515 in Europe, 0.3917 in Oceania, 0.389 in Latin America and Caribbean, to 0.339 in Asia and 0.202 in Africa. By HDI, there was a significant trend of increase in proxy SS-RS from a median of 0.188 in the “low” and 0.295 in the “medium” tiers, to 0.391 in the “high” and 0.540 in the “very high” tiers (Fig. 3). All three categorizations showed significant variations among the different subgroups (*P* < 0.001).

### Correlation between cancer site-standardized proxy RS and economic indicators

**Fig. 4:**
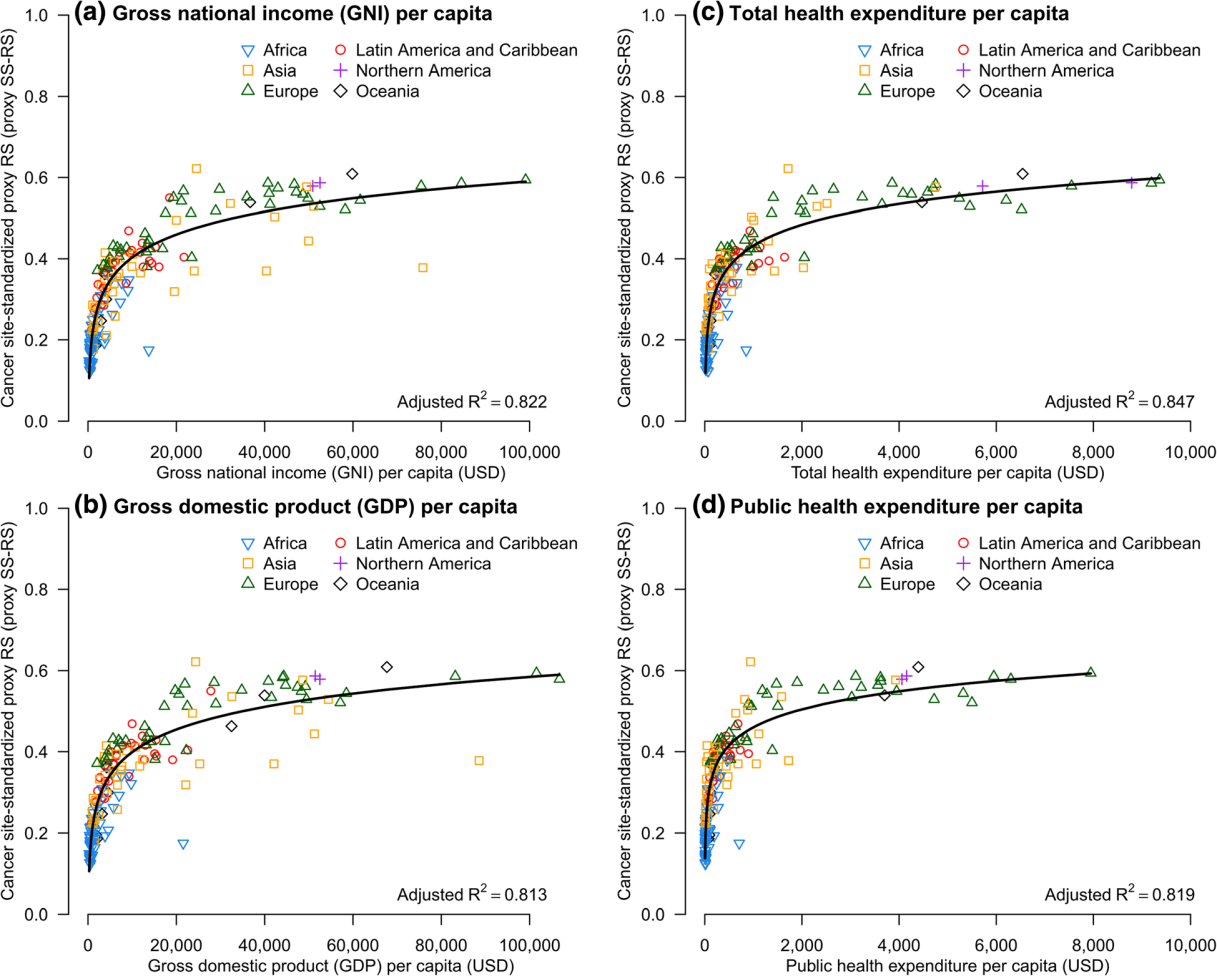
Cancer site-standardized proxy relative survival (proxy SS-RS) against economic indicators. **a** gross national income per capita, GNIpc; **b** gross domestic product per capita, GDPpc; **c** total health expenditure per capita, total HEpc; and **d** public health expenditure per capita, public HEpc

Figure 4 shows the patterns of correlation between proxy SS-RS and economic indicators, and the fitted lines by logarithmic regression models. The plots showed significant correlation with high adjusted R^2^ ≥0.8 for the four economic indicators studied (*P* < 0.001). Total HEpc was the best fitted indicator with the highest adjusted R^2^ of 0.847 (Additional file 1: Table S2). Table 3a shows the actual monetary values recorded in countries by proxy SS-RS. The median actual total HEpc increased from US$44 among countries with proxy SS-RS <0.25, US$183 for SS-RS 0.25 − <0.35, US$551 for SS-RS 0.35 − <0.45, US$2189 for SS-RS 0.45 − <0.55, to US$4643 for countries with SS-RS ≥0.55. The actual expenditure also varied widely within each range of proxy SS-RS: in particular, among the 19 countries with proxy SS-RS ≥0.55, the actual total HEpc ranged widely from US$1412 to US$9361; the expenditure in the top 3 countries with the highest proxy SS-RS was: Republic of Korea (US$1715), Australia (US$6544) and Norway (US$9196) (Table 1).

**Table 3:** Pattern between economic indicators and site-standardized proxy relative survival (proxy SS-RS)

SS-RS	GNIpc	GDPpc	Total HEpc	Public HEpc
(a) Recorded actual monetary values (US$) by proxy SS-RS [median (range)]				
0.124 – <0.25	830 (260–13,760)	903 (265–21,558)	44 (15–853)	16 (7–709)
0.25 – <0.35	3500 (1040–19,560)	3737 (955–22,135)	183 (61–674)	101 (18–478)
0.35 – <0.45	8450 (2140–75,850)	8558 (2047–88,565)	551 (93–2049)	320 (36–1726)
0.45 – <0.55	34,480 (9210–61,650)	32,570 (9985–58,508)	2189 (931–6522)	1392 (629–5501)
0.55 – ≤0.622	46,710 (18,460–99,100)	44,741 (19,730–106,749)	4643 (1412–9361)	3590 (942–7947)
Targeted SS-RS	GNIpc	GDPpc	Total HEpc	Public HEpc
(b) Projected monetary value (US$) [mean (95% confidence interval)] needed to reach a target proxy SS-RS				
0.25	1524 (1322–1736)	1567 (1350–1795)	85 (74–97)	38 (31–44)
0.35	5238 (4713–5823)	5447 (4880–6081)	328 (295–365)	178 (156–204)
0.45	18,003 (15,774–20,810)	18,930 (16,497–22,025)	1260 (1104–1454)	846 (716–1016)
0.55	61,874 (50,963–77,034)	65,792 (53,743–82,776)	4840 (3989–6005)	4013 (3141–5291)

Abbreviations: *GDPpc* Gross domestic product per capita, *GNIpc* Gross national income per capita, *Total HEpc* Total health expenditure per capita, *public HEpc* Public health expenditure per capita

Economic monetary values were rounded to integers

The best data fit with logarithmic regression model showed exponential increase in economic indicators with improving proxy SS-RS, with marked flattening of the slope for changes among countries with proxy SS-RS ≥0.5 (Fig. 4). The estimated total HEpc associated with a proxy SS-RS of 0.35, 0.45 and 0.55 were US$328, US$1260, and US$4840, respectively (Table 3b). The projected additional total HEpc needed to achieve an increment of 0.1 in proxy SS-RS increased exponentially from US$932 to US$3580 for improving proxy SS-RS from 0.35 to 0.45 and from 0.45 to 0.55, respectively (Table 3b).

## Discussion

Cancer is one of the most serious health problems globally. Four major international studies on comparisons of cancer survival have been published in the recent decade. They provided comprehensive epidemiological data in different cancer types among selected countries and their population-based registries [9–12]. The CONCORD-3 study has recently been published to include even more population-based registries in more countries [13]. These landmark reports provide insight on global cancer landscapes from different perspective but the interpretation of these results is hampered by the wide variations in cancer pattern and thus survival outcomes. To the best of our knowledge, this is the first report of comprehensive survival estimates that took into account the pattern of cancer of all countries in the world. To complement the research gap, the current paper introduced the concept of cancer site-standardization with adjustment for wide variation in distribution of different cancer sites across different countries. It incorporated relatively simple and reproducible methodology using GLOBOCAN data as the best publicly accessible and creditable sources. Such standardization potentially helps develop more refined cross-country comparisons for the assessment of cost-efficiency of different healthcare systems.

It is well recognized that age-standardization of incidence and mortality rates are needed for comparison of cancer burden across different countries and across different time periods for the same country because this vital factor varies widely with time for different populations and sociohistorical indicators could also contribute to the variations across time horizon [14]. Following the same principle, given the marked differences in incidence patterns and survival outcomes, proxy SS-RS which adjusts for variation in cancer site distribution is recommended for cross-country comparisons, especially for studies aiming at sharing of experience on cost-efficiency of health systems in different countries.

We showed significant correlation between proxy SS-RS and all the economic indicators studied. The magnitude of exponential increase in health expenditure required to achieve the highest SS-RS was striking: the estimated total HEpc associated with a proxy SS-RS of 0.35, 0.45 and 0.55 would be as high as US$328, US$1260 and US$4840, respectively (Table 3b). In fact, the cancer burden is adversely affecting not only those resource-constrained countries, but also those more effluent and developed countries alike [15–18]. For the low- and middle-income countries (LMICs; GNI per capita <US$1035 and <US$4085 in 2012, respectively), increasing HEpc to improve access to essential treatment is crucial. Unfortunately, more than 80% LMICs failed to afford such expenditure. Innovative financing, partnership between private sector and international development banks will be useful for sustainable financing in resource-constrained countries.

Global disparity in access to systemic therapy is a well-known phenomenon [19–21]. The expanding list of novel agents including chemotherapy, target therapy and immunotherapy will only, in a vicious circle, cause escalating cost of treatment, wider disparity in access and thus variations in treatment outcomes. Similarly, the access to radiotherapy (RT), an essential component of cancer treatment, is especially poor in many LMICs due to the apparently high capital cost. The recent study by Lam et al. showed a strong correlation between treatment outcomes of nasopharyngeal carcinoma (a highly curable cancer for which RT is the primary treatment modality) and the availability of both radiotherapy facilities and radiation oncologists [22]. Unfortunately, 80% of new cases occurred in countries with limited access to RT and thus worse outcomes. The study by Atun et al. demonstrated the substantial benefit to investing in RT, advocating concerted global effort to expand RT access and to develop financially sustainable population-based cancer control plans [23]. Furthermore, cancer control ranging from prevention, screening, cancer treatment and palliative care are also crucial for better cancer outcomes. For example, cervical screening contributed to lower mortality by screening early stage, and more curable, cancer cases. The cost-effectiveness of implementing human papillomavirus (HPV) genotyping to detect the infection of high-risk oncogenic HPV types and the frequency of follow-up screening are, however, subjective to the local disease patterns, healthcare infrastructure and affordability and should be studied carefully for each individual country [24, 25]. Taking all these together, a standardized indicator of cancer outcomes that adjusted for patterns of cancer in each individual country will be invaluable for developing a more cost-efficient cancer plan from prevention to prioritizing treatment access with the resources available.

Consistent with the findings of escalating health expenditures in our study, thus far there are a number of reports on financial burden caused by cancer in high income countries with complex estimations. The study focused on countries in the European Union showed that the total cost amounted to approximately US$176 billion in 2009 (including US$71 billion direct expenditure on health care services, and indirect cost of productivity losses US$18 billion due to early death and US$13 billion due to lost working day) [26]. The estimated drug cost on cancers increased markedly from US$9.5 billion in 2005 to US$25.4 billion in 2014 [27]. Similarly, the study in the United States showed alarming cost on cancer care amounting to US$137 billion in 2014 [28]. With these escalating expenditures, financial hardship at personal and household level are anticipated despite medical insurance coverage. According to the survey from the Health and Retirement Study in the United States [29], which included 1409 cancer patients who were all community-dwelling Medicare beneficiaries, 24–63% of patients’ household income were spent on health care; the proportion due to cancer was substantially higher than non-cancer disease (adjusted odds ratio, 1.86; 95% CI, 1.55–2.23). Even in countries where access to therapy is not the main problem, the huge cost incurred will adversely affect sustainable affordability [30]. Our proxy SS-RS can be applied as the reference indicators for comparing outcomes and cost-efficiencies of different countries from time-to-time whenever relevant economic indicators are updated.

Our study has the following limitations. The cancer statistics are based on data from GLOBOCAN 2012: the quality of data is suboptimal in many of the LMICs and more updated data will be desirable, although the principle of standardization should still hold true. The survival outcome used was a surrogate calculated using the formula [1 – (ASM/ASI)] rather than the more desirable but currently unavailable prospective data from most cancer registries using age, sex, stage at presentation and treatment exposures. The monetary values on expenditures available from the World Bank only provide data on all health care, but not specific expenditure attributed to cancer. However, the study in the European Union showed that the proportion of total HEpc spent on cancer-specific care was fairly constant among countries, hence the total HEpc used in the current study is a reasonable proxy for comparison [27]. It would be a mammoth task to incorporate all potential confounders for a real world analysis. Factors like the cost of living index and purchasing power parity were unavailable for our study but it may be explored in future studies for optimizing global comparison.

Nonetheless, standardization of RS minimizes the bias in comparison across all listed countries basing on best available data. The findings are useful for provoking greater awareness to the global variation in treatment outcomes for cancer patients and suggesting a strong link to the investment in health care. It is a huge challenge to policy makers to develop a comprehensive cancer plan that is cost-efficient and financially sustainable. This fundamental need require urgent improvement as the economic burden of cancer is so huge and so rapidly escalating for all countries. It is hoped that our study will stimulate more in-depth studies to overcome the existing limitations, to provide essential data for sharing of experience and to assist the global communities to make a concerted effort towards the commitment of reducing cancer mortality as stated in the World Health Assembly Cancer Resolution.

## Conclusions

Adjustment for variation in incidence of different cancer with different chance of cure by standardization is advocated for cross-country comparisons of cancer outcomes. The correlation of proxy SS-RS with national economic indicators is strongly significant and that the findings of global disparity is striking. Our findings call for more in-depth studies to provide essential data for sharing of experience and urgent actions by policy makers to develop a comprehensive cancer plan that is cost-efficient and financially sustainable.

## Additional file

Additional file 1: **Table S1.** Calculation of site-standardized proxy RS (proxy SS-RS). **Table S2.** Regression coefficients of site-standardized proxy relative survival (proxy SS-RS) on economic indicators. (DOCX 26 kb)

## Data Availability

All data generated or analysed during this study are included in this published article and its supplementary materials.

## Abbreviations

ASI: Age-standardized rates of incidence
ASM: Age-standardized rates of mortality
CI: Confidence interval
GDP: Gross domestic product
GNI: Gross national income
HDI: Human Development Index
HEpc: Health expenditure per capita
IARC: International Agency for Research on Cancer
LMICs: Low- and middle-income countries
Proxy SS-RS: Site-standardized proxy RS
RS: Relative survival
RT: Radiotherapy
UICC: Union for International Cancer Control
