# Supplementary material for: Global comparison of cancer outcomes: standardization and correlation with healthcare expenditures
Source: BMC Public Health. 2019 Aug 7;19:1065. doi: 10.1186/s12889-019-7384-y (PMC6686500; doi:10.1186/s12889-019-7384-y)
Supplement: Supplementary file 1 — Table S1. Calculation of site-standardized proxy RS (proxy SS-RS). Table S2. Regression coefficients of site-standardized proxy relative survival (proxy SS-RS) on economic indicators. (DOCX 26 kb) [file 12889_2019_7384_MOESM1_ESM.docx]

# Additional file 1

**Global comparison of cancer outcomes: standardization and correlation with healthcare expenditures.**

Horace CW Choi^1^, Ka-On Lam^* 1,2^, Herbert HM Pang^3^, Steven KC Tsang^1^, Roger KC Ngan^1^, Anne WM Lee^1,2^

^1^ Department of Clinical Oncology, Li Ka Shing Faculty of Medicine, The University of Hong Kong

^2^ Clinical Oncology Center, The University of Hong Kong-Shenzhen Hospital, Shenzhen, China

^3^ School of Public Health, Li Ka Shing Faculty of Medicine, The University of Hong Kong

* Corresponding author

Email: lamkaon@hku.hk

**Table S1 . Calculation of site-standardized proxy RS (proxy SS‑RS).**

|  | **World** | | **Country A** | | | |
| --- | --- | --- | --- | --- | --- | --- |
| **Cancer sites** | **Incidence (a)** | **% (b)** | **ASI (c)** | **ASM (d)** | **Site-specific proxy RS (e)** | **Site-standardization (f)** |
| **Bladder** | 429,793 | 3·3 | 7·6 | 2·0 | 0·737 | 2·43 |
| **Brain, nervous system** | 256,213 | 2·0 | 5·4 | 3·9 | 0·278 | 0·56 |
| **Breast** | 1,671,149 | 12·9 | 86·0 | 14·0 | 0·837 | 10·80 |
| **Cervix uteri** | 527,624 | 4·1 | 5·5 | 1·6 | 0·709 | 2·91 |
| **Colorectum** | 1,360,602 | 10·5 | 38·4 | 9·0 | 0·766 | 8·04 |
| **Corpus uteri** | 319,605 | 2·5 | 12·1 | 1·4 | 0·884 | 2·21 |
| **Gallbladder** | 178,101 | 1·4 | 1·5 | 0·5 | 0·667 | 0·93 |
| **Hodgkin lymphoma** | 65,950 | 0·5 | 2·4 | 0·2 | 0·917 | 0·46 |
| **Kaposi sarcoma** | 44,247 | 0·3 | 0·2 | 0·0 | 1·000 | 0·30 |
| **Kidney** | 337,860 | 2·6 | 9·5 | 2·1 | 0·779 | 2·03 |
| **Larynx** | 156,877 | 1·2 | 1·7 | 0·5 | 0·706 | 0·85 |
| **Leukaemia** | 351,965 | 2·7 | 9·4 | 3·5 | 0·628 | 1·70 |
| **Lip, oral cavity** | 300,373 | 2·3 | 6·3 | 1·0 | 0·841 | 1·93 |
| **Liver** | 782,451 | 6·0 | 4·2 | 3·7 | 0·119 | 0·71 |
| **Lung** | 1,824,701 | 14·0 | 27·0 | 18·5 | 0·315 | 4·41 |
| **Melanoma of skin** | 232,130 | 1·8 | 34·9 | 4·0 | 0·885 | 1·59 |
| **Multiple myeloma** | 114,251 | 0·9 | 3·7 | 1·9 | 0·486 | 0·44 |
| **Nasopharynx** | 86,691 | 0·7 | 0·4 | 0·2 | 0·500 | 0·35 |
| **Non-Hodgkin lymphoma** | 385,741 | 3·0 | 12·2 | 3·0 | 0·754 | 2·26 |
| **Oesophagus** | 455,784 | 3·5 | 3·5 | 2·9 | 0·171 | 0·60 |
| **Other pharynx** | 142,387 | 1·1 | 2·0 | 0·7 | 0·650 | 0·72 |
| **Ovary** | 238,719 | 1·8 | 7·6 | 4·4 | 0·421 | 0·76 |
| **Pancreas** | 337,872 | 2·6 | 6·6 | 5·6 | 0·152 | 0·40 |
| **Prostate** | 1,094,916 | 8·4 | 115·2 | 12·9 | 0·888 | 7·46 |
| **Stomach** | 951,594 | 7·3 | 4·8 | 2·5 | 0·479 | 3·50 |
| **Testis** | 55,266 | 0·4 | 6·6 | 0·1 | 0·985 | 0·39 |
| **Thyroid** | 298,102 | 2·3 | 8·4 | 0·3 | 0·964 | 2·22 |
| **All cancers excluding non-melanoma skin cancer** | | | 323·0 | 96·4 | 0·702 |  |
| **Site-standardized proxy RS (proxy SS‑RS)** | | |  |  |  | ÷ 100 = 0·609 |

Abbreviations: ASM, age-standardized rate of mortality; ASI, age-standardized rate of incidence; RS, relative survival.

Site-standardized proxy RS (f) = sum( column (b) × column (e) ) ÷ 100 = for *i*=each cancer site.

**Table S2 . Regression coefficients of site-standardized proxy relative survival (proxy SS‑RS) on economic indicators.**

| **Economic indicators (per capita)** | **Regression coefficient for *ln*(indicator)** | **Change to response by % increase in indicators ^b^** | | |  |
| --- | --- | --- | --- | --- | --- |
|  |  | **10% increase** | **25% increase** | **50% increase** | **adjR^2^** |
| **Response: Site-standardized proxy RS (proxy SS‑RS) ^a^** | | | | | |
| **GNIpc** | 0·081  (0·075, 0·087) | 0·0077  (0·0071, 0·0082) | 0·018  (0·017, 0·019) | 0·033  (0·030, 0·035) | 0·819 |
| **GDPpc** | 0·080  (0·074, 0·086) | 0·0076  (0·0070, 0·0082) | 0·018  (0·016, 0·019) | 0·032  (0·030, 0·035) | 0·808 |
| **Total HEpc** | 0·074  (0·069, 0·079) | 0·0071  (0·0066, 0·0075) | 0·017  (0·016, 0·018) | 0·030  (0·028, 0·032) | 0·847 |
| **Public HEpc** | 0·064  (0·060, 0·067) | 0·0061  (0·0057, 0·0066) | 0·014  (0·013, 0·015) | 0·026  (0·024, 0·028) | 0·819 |

Abbreviations: adjR^2^, adjusted R-squared; GDPpc, gross domestic product per capita; GNIpc, gross national income per capita; HDI, Human Development Index; public HEpc, public health expenditure per capita; RS, relative survival; total HEpc, total health expenditure per capita.

**^a^** Each cell presents the point estimate and 95% confidence interval.

**^b^** Note: By the property of logarithm, 1% increase in economic indicator would result in changing response (cancer site-standardized proxy RS) by *ln*(1·01)×*β* unit (i.e. approximately 0·01×*β* unit), with *β* refers to the corresponding regression coefficient. Because of the different interpretation from ordinary linear regression models and to better illustrate the results, the influence to RS given 10%, 25% and 50% relative increase in the economic indicators for the logarithmic regression models were presented.
